# The temporal stability of core symptoms of social media addiction and their comorbidity with anxiety and depression in adolescents: a longitudinal network analysis

**DOI:** 10.3389/fpsyt.2026.1785472

**Published:** 2026-04-22

**Authors:** Wenxin Xu, Yu Huang, Chi Su, Zhibin Zhou, Shiying Wang, Haolin Ye, Yueshan Xu, Yanli Wang, Kezhi Liu, Jing Chen, Wei Lei

**Affiliations:** 1 Department of Psychiatry, the Affiliated Hospital of Southwest Medical University, Luzhou, China; 2 Fundamental and Clinical Research on Mental Disorders Key Laboratory of Luzhou City, Luzhou, China; 3 School of Clinical Medicine, Southwest Medical University, Luzhou, China

**Keywords:** anxiety, depression, longitudinal study, network analysis, social media addiction

## Abstract

**Introduction:**

Social media addiction (SMA) is often comorbid with anxiety and depression. This study examined the temporal stability of core SMA symptoms and the bridging symptoms with anxiety and depression.

**Methods:**

A total of 1,240 adolescents (179 males, 1,061 females; mean age = 15.46 ± 0.63 years, age range: 14 – 18) completed the Bergen Social Media Addiction Scale (BSMAS), the Patient Health Questionnaire–9 (PHQ–9), and the Generalized Anxiety Disorder–7 (GAD–7) on two separate occasions in 2023 (T1) and 2024 (T2). The four symptom networks, including the BSMAS networks, two comorbidity networks (the BSMAS–GAD and the BSMAS–PHQ), and the integrated BSMAS–GAD–PHQ network, were estimated using Gaussian graphical models. Core symptom centrality was assessed using Expected Influence (EI), whereas bridge symptoms were identified using Bridge Expected Influence (BEI).

**Results:**

1) Although SMA, anxiety, and depression levels of respondents rose significantly over the year, all four networks showed strong temporal stability, with the edge weights (*r* = .892 –.973, *p* < .001), the EI (*r* = .806 – .961, *p* ≤ .002), and the BEI (*r* = .699 – .804, *p* ≤ .008) highly correlated between T1 and T2; network comparison tests showed no significant changes in overall structures of all four networks, with most edges showing stable weights. 2) Within the BSMAS network, BSMAS2 (tolerance) and BSMAS6 (conflict) exhibited the highest EI at both time points. 3) In the comorbidity networks, BSMAS3 (mood modification), BSMAS5 (withdrawal), and BSMAS6 (conflict) consistently served as bridge symptoms on the SMA side at both T1 and T2. 4) Across both time points, PHQ1 (anhedonia) and PHQ7 (concentration problems) exhibited the highest BEI on the depression side, whereas GAD1 (nervousness) and GAD5 (restlessness) did so on the anxiety side. 5) These bridge symptoms were also confirmed in the integrated network.

**Discussion:**

These findings illuminate the temporal persistence and development of symptom relationships, offering a more dynamic understanding of SMA–depression–anxiety comorbidity in adolescents.

## 1 Introduction

Since the early 2000s, social media platforms such as Facebook, Instagram, TikTok, and Weibo have transformed global communication and information sharing. As of 2025, approximately 5.56 billion people used the Internet, with 88% (4.9 billion) of them actively engaging on social media (1). Excessive use has raised concerns about social media addiction (SMA), characterized by compulsive social media use that disrupts academic, social, or personal functioning (2, 3). A meta-analysis reported a prevalence of 5% (strict criteria) to 25% (lenient criteria) for SMA (4).

Adolescence is a critical developmental period for the emergence of mental disorders. According to recent global estimates reported by the World Health Organization, anxiety disorders affect 4.1% of adolescents aged 10–14 years and 5.3% of those aged 15–19 years, whereas depressive disorders affect 1.3% and 3.4% of these age groups, respectively (5). Addictive disorders are highly comorbid with anxiety disorders and depression (6–8). This pattern of comorbidity also applies to SMA—in youth populations, consistent positive correlations have been documented between SMA and symptoms of depression and anxiety (9–11). A scoping review of 43 studies conducted by Azem et al. (12) indicated that adolescents’ social media use, encompassing usage time, frequency, and problematic use, was associated with multiple mental health problems, including depressive symptoms, anxiety, sleep problems, diminished self-esteem, etc. Prior research has proposed that SMA may adversely affect adolescents’ mental health through mechanisms such as sleep deprivation, social comparison, and cyberbullying (13, 14). Similarly, a recent person-centered analysis revealed that distinct patterns of social media use were differentially associated with depressive symptoms, underscoring the role of SMA in vulnerability for depression among adolescents (15). A systematic review by Lopes et al. (16) suggested a bidirectional link between SMA and depression. In addition, because depression and anxiety themselves exhibit reciprocal relationships (17), their mutual reinforcement may further amplify the interconnectedness of SMA and internalizing symptoms, creating a self-sustaining cycle of emotional dysregulation and problematic social media engagement. Conversely, adolescents already experiencing depression or anxiety may turn to social media for comfort or avoidance, which in turn increases the risk of problematic use. Two recent studies demonstrated this perspective. Shannon et al. (18) found that increases in SMA severity in students were associated with subsequent elevations in depression, anxiety, and stress symptoms, while Nagata et al. (19) reported that higher social media use in early adolescence predicted greater depressive symptoms at follow-up.

In recent years, network analysis has become a valuable tool for understanding the structure and development of psychological symptoms. This method sees emotions, thoughts, and behaviors as “nodes,” and the connections between them as “edges,” building a network of the mental system. Unlike traditional methods, network analysis focuses on how these symptoms are related and how they affect each other (20, 21). Beyond descriptive mapping, network analysis holds unique advantages: it can clarify mechanisms of comorbidity (22), identify core symptoms or bridging symptoms as promising intervention targets (23). Bridging symptoms refer to symptoms that link different disorders, and may play a key role in how disorders appear together (24, 25). Some studies have examined the relationship between SMA, depression, and anxiety, explored bridge symptoms linking these conditions, using network analysis (26–30). For example, Li et al. (27) found that the SMA symptoms “withdrawal” and “conflict” acted as key bridge symptoms linking SMA with psychological distress (anxiety, depression, and stress), meaning in life, Internet gaming disorder (IGD), and problematic smartphone use. Similarly, Yang et al. (29) reported that SMA symptoms such as “mood modification” and “conflict” acted as bridges in a comorbidity network including SMA, depression, and anxiety. Two recent studies also stressed the interrelations between SMA, anxiety, and depression and their combined impact on participants’ mental health (31, 32).

Importantly, comorbidity between behavioral addictions and psychological symptoms may be dynamic. Longitudinal network models can reveal how symptoms fluctuate over time and which remain stable, offering insights into the onset, persistence, and development of mental health problems (23, 33–35). Peng et al. (36) used growth mixture modeling to identify four IGD trajectories and showed that as addictive behaviors escalate, the expression and importance of bridge symptoms—such as sleep disturbance, irritability, and attentional problems—that mediate links between behavioral addictions and internalizing symptoms (e.g., negative emotions) may change. According to the Interaction of Person-Affect-Cognition-Execution (I-PACE) model of behavioral addiction (37), behavioral addictions like SMA are characterized by affective dysregulation and excessive negative emotions, including depression and anxiety. From this theoretical standpoint, changes in addiction severity may occur alongside shifts in the bridge symptoms that connect SMA with psychological distress. Longitudinal studies further support this temporal dynamic. For example, the studies of Shannon et al. (18) and Nagata et al. (19) suggest that the interaction between social media use, depression, and anxiety may depend on the initial symptoms that affected the subject. Moreover, Tullett-Prado, et al. (30) applied longitudinal network analysis to show that specific SMA symptoms assumed key roles in symptom networks, indicating that the interactions between SMA and psychological distress may shift over time. Moreover, Ahmed et al. (38) identified sleep problems as a partial mediator in the longitudinal associations between SMA and both depression and anxiety. However, most existing studies focus on total scores or use cross-sectional designs, which cannot uncover the directional links or feedback loops between specific symptoms. There remains a lack of systematic research on how individual symptoms interact within a network and how such networks evolve over time.

This study aims to examine the core symptoms of SMA and the bridging symptoms linking SMA with depression and anxiety, as well as their stability across time, using network analysis. The findings may help identify potential intervention targets for adolescents with SMA.

## 2 Methods

### 2.1 Participants and procedure

The survey was administered in October 2023 and October 2024 to students at a secondary nursing school in Luzhou, China. The survey link was distributed via a QR code posted in class messaging groups. Because the cohort was predominantly female, the sample was correspondingly skewed toward women; this was considered acceptable given evidence that women are more likely than men to exhibit social media addiction (39). Before participation, all students were informed about the study’s objectives and procedures, and were invited to take part with consent from their guardians. Informed consent was obtained at the beginning of the survey: the first item asked participants to either agree to continue or opt out. They were also reminded that they could discontinue the survey at any time without consequences. Respondents received additional course credit for completing the survey. The study protocol was approved by the Ethics Committee of the Affiliated Hospital of Southwest Medical University (Approval No. KY2024181).

A total of 1,980 adolescents participated in the first wave of the survey, with 1,336 of them also taking part in the second wave. During sample screening, participants were excluded if they 1) did not complete both surveys (*n* = 644); 2) provided an impossible age (e.g., reported an age of 99, *n* = 197); or 3) reported a personal history of mental disorders (*n* = 24). All survey items were mandatory, so respondents could not skip individual questions, although they were free to withdraw at any time. No respondents quit the survey midway. After applying the screening criteria, 1,240 students who provided valid responses in both waves were retained for further analysis.

### 2.2 Measurements

Demographic information was collected using a home-designed questionnaire that asked about age, gender, grade, and history of mental disorders. These items were used to characterize the sample and to screen for valid respondents.

#### 2.2.1 Bergen social media addiction scale

The BSMAS was used to assess symptoms of social media addiction (40). The scale has six items rated from 1 (almost never) to 5 (very often). Each item represents one symptom of SMA, including salience, tolerance, mood modification, relapse, withdrawal, and conflict. A total score of 19 or higher indicates possible addiction (41). In this study, the Cronbach’s alpha of the BSMAS was.842 at baseline (T1) and.885 at follow-up (T2) survey.

#### 2.2.2 Patient health questionnaire–9

The PHQ–9 was used to assess depressive symptoms over the past two weeks in respondents (42, 43). The scale has 9 items rated on a Likert scale of 0 (not at all) to 3 (nearly every day). The Chinese version of the PHQ–9 has been shown to have good validity and reliability (44). In this study, the Cronbach’s alpha of the PHQ–9 was.921 at T1 and.930 at T2.

#### 2.2.3 Generalized anxiety disorder–7

The GAD–7 was used to assess anxiety symptoms over the past two weeks (45). It includes 7 items rated on a 4-point Likert scale from 0 (not at all) to 3 (nearly every day). The GAD–7 has been widely used and validated in Chinese adolescents (46). In this study, the Cronbach’s alpha of the GAD–7 was.925 at T1 and.932 at T2.

### 2.3 Statistical analysis

#### 2.3.1 Descriptive and longitudinal comparisons

Descriptive statistics for all variables were calculated using SPSS 26.0. To examine changes over time, paired t-tests were conducted to compare the scale scores between T1 and T2. Furthermore, Pearson’s correlations were performed to assess the temporal stability of the associations among these scale scores. All statistical tests were two-tailed, and significance was set at *p* <.05.

### 2.4 Network analysis

Symptom networks were estimated using the bootnet (v1.5.3) package in R (version 4.4.1) (47). A total of 22 ordinal items from three scales were included: the BSMAS (6 items), the PHQ–9 (9 items), and the GAD–7 (7 items). Pairwise polychoric correlations were computed using the cor_auto function, which is appropriate for ordered categorical data (48). Networks were estimated via the EBICglasso method with a tuning parameter of *γ* = 0.5. No fixed edge-weight threshold was applied. Networks were visualized with qgraph (v1.9.8) (48, 49), with nodes color-coded by scale to distinguish symptom groups.

Network accuracy and robustness were evaluated using the bootnet package. A nonparametric bootstrap was used to estimate edge-weight accuracy, and a case-dropping bootstrap was used to assess the stability of centrality estimates (47). The correlation Stability (CS) coefficient was calculated as an index of centrality stability; CS ≥.25 was considered acceptable and CS ≥.50 excellent (47). All bootstrapping processes were conducted 1000 times as recommended (S. Epskamp et al., 2018). Finally, the difference test function was used to compare edge weights and centrality metrics within networks.

#### 2.4.1 Network structure

At both time points, four networks were estimated: the BSMAS network, two comorbidity networks (the BSMAS–PHQ network and the BSMAS–GAD network), and the integrated BSMAS–PHQ–GAD network. For the BSMAS network, node centrality was assessed using Expected Influence (EI). EI is defined as the sum of all edge weights connected to a node, which has been shown to be more reliable in psychopathology networks than other centrality metrics such as closeness and betweenness (50). For the comorbidity and integrated networks, bridge symptoms were identified using Bridge Expected Influence (BEI). BEI quantifies a node’s total connectivity to nodes in other symptom clusters while preserving the sign (positive or negative) of edge weights. Previous research has shown that BEI is more robust than bridge strength in the presence of negative edges, making it a better indicator of nodes that are critical for symptom transmission across clusters (23, 24).

#### 2.4.2 Network temporal stability analysis

To evaluate the temporal stability of the node-level metrics, Pearson’s correlations were calculated between corresponding edge weights and centralities across the two time points. Correlations in edge weights were used to reflect similarity in the overall network structure, whereas correlations in EI and BEI were used to reflect similarity in node importance across time. Statistical significance was set at *p* <.05.

To further assess the topological consistency of the overall network structures, the Network Comparison Test (NCT) was conducted between T1 and T2 for each of the four networks. This analysis was performed using the NCT package in R (51).

## 3 Results

### 3.1 Descriptive statistics

The final sample consisted of 1,240 students, including 179 boys and 1,061 girls, with a mean age of 15.46 ± 0.63 years (range: 14–18 years). The mean scores of the BSMAS, the PHQ–9, and the GAD–7 all showed a significant increase from T1 to T2 (all *p* <.001; **Table 1**). Nevertheless, paired correlation analyses indicated that all three scales demonstrated moderate-to-high temporal stability according to Cohen’s (52) guidelines, with Pearson correlation coefficients ≥.457 (all *p* <.001; **Table 2**). Furthermore, the correlation matrix of total scores across both time points (T1 and T2) showed significant positive associations among the BSMAS, the PHQ–9, and the GAD–7 (*r* = .252 –.748, all *p* <.001), indicating significant mutual associations between these symptom sets (**Table 2**).

**Table 1:** Paired-samples t-tests of SMA, depression, and anxiety symptoms at T1 and T2.

Network	T1	T2	*t*	*p*
BSMAS	10.580 ± 3.773	11.120 ± 4.136	-4.541	<.001
PHQ–9	5.370 ± 4.777	6.310 ± 5.062	-6.675	<.001
GAD–7	4.800 ± 3.920	5.200 ± 4.077	-3.584	<.001

Data are presented as *M ± SD*; All *p-values* are two-tailed.

**Table 2:** Pearson’s Correlations among total scores of the BSMAS, the PHQ–9, and the GAD–7 across two time points.

Network	BSMAS T1	BSMAS T2	GAD–7 T1	GAD–7 T2	PHQ–9 T1
BSMAS T1	–				
BSMAS T2	.457^**^	–			
GAD–7 T1	.398^**^	.329^**^	–		
GAD–7 T2	.252^**^	.431^**^	.514^**^	–	
PHQ–9 T1	.389^**^	.304^**^	.748^**^	.478^**^	–
PHQ–9 T2	.290^**^	.436^**^	.491^**^	.748^**^	.502^**^

All *p-values* are two-tailed. ^**^p <.01, ^*^p <.05.

### 3.2 The BSMAS network

**Figure 1:**
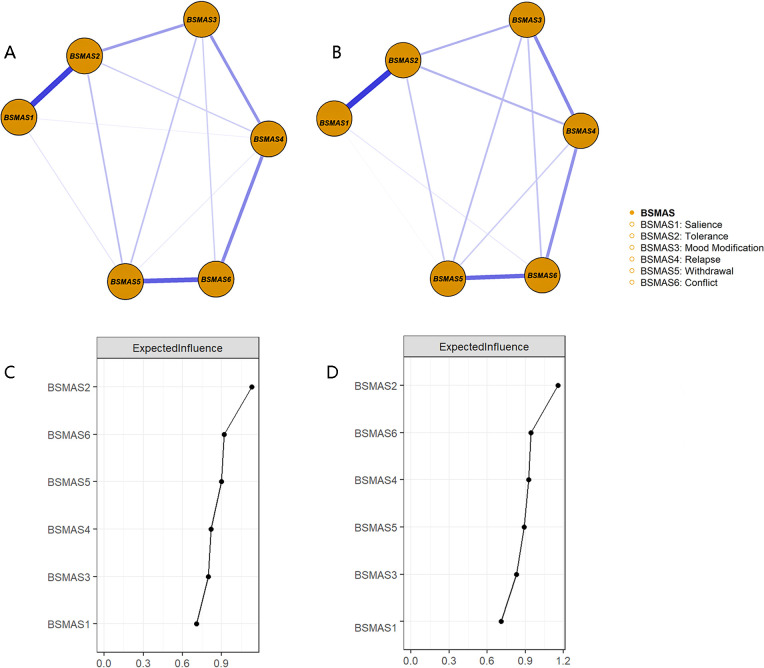
The symptom network structure and the EI centrality plots for the BSMAS network. The symptom networks for the BSMAS at T1 **(A, C)** and T2 **(B, D)**. Orange nodes represent individual symptoms. Edge thickness reflects the strength of associations; solid lines denote positive correlations and dashed lines indicate negative correlations. Node labels refer to specific items.

At both T1 and T2, the BSMAS2 (tolerance, the EI = 1.130 at T1 and the EI = 1.155 at T2) and BSMAS6 (conflict, the EI = 0.920 at T1 and the EI = 0.945 at T2) were consistently identified as the core symptoms of SMA (**Figure 1**). Difference tests indicated that BSMAS2 showed significantly higher EI than other symptoms, while BSMAS6 showed numerically higher EI values than the remaining symptoms (**Supplementary Figure 1**). Edge-weight difference tests confirmed that edges involving BSMAS2 and BSMAS6—especially the BSMAS1–BSMAS2 and BSMAS5–BSMAS6—were stronger than other edges (see **Supplementary Figure 2**). Edge weight and centrality stability at both time points were excellent with CS ≥.75 (**Supplementary Table 1**, **Supplementary Figure 3**).

### 3.3 The BSMAS–GAD comorbidity network

**Figure 2:**
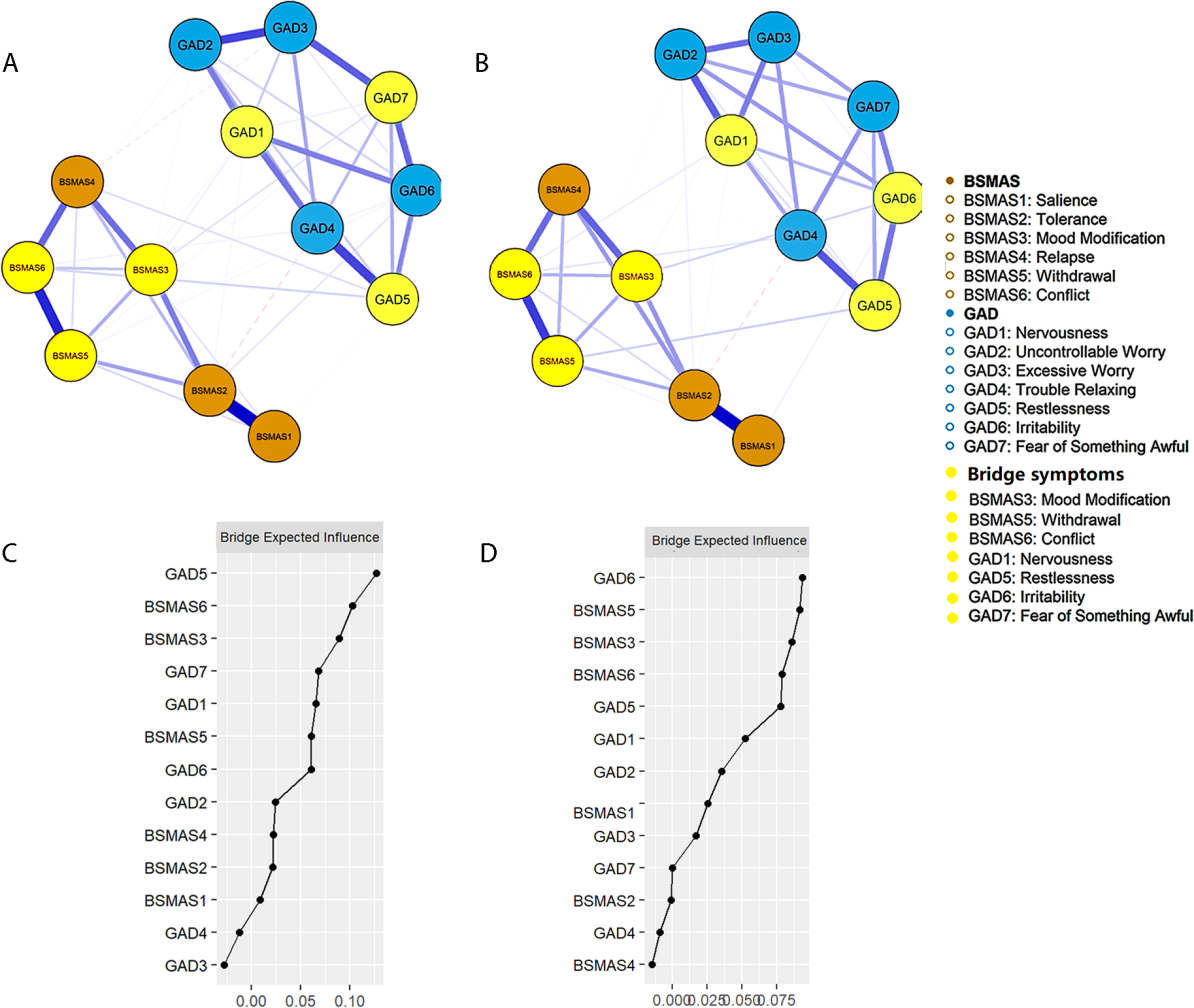
The comorbidity network structure and the BEI centrality plots for the BSMAS–GAD network. The symptom networks for the BSMAS and the GAD–7 at T1 **(A, C)** and T2 **(B, D)**. Orange nodes represent the BSMAS symptoms, blue nodes represent the GAD–7 symptoms, and yellow nodes indicate bridge symptoms. Edge thickness reflects partial correlations between symptoms; solid lines indicate positive associations, and dashed lines indicate negative associations. Node labels correspond to specific scale items as defined in the main text.

The BSMAS3 (mood modification), BSMAS5 (withdrawal), and BSMAS6 (conflict) were consistently identified as bridging symptoms on the SMA side across both time points (**Figure 2**). Among the anxiety symptoms, GAD5 (restlessness), GAD7 (fear of something awful), and GAD1 (nervousness) were recognized as bridging symptoms at T1. At T2, GAD6 (irritability), GAD5 (restlessness), and GAD1 (nervousness) were identified as bridging symptoms. Difference tests showed that the bridge symptoms on both sides had significantly higher BEI values than other symptoms across the two time points (**Supplementary Figure 4**). Edge-weight difference tests indicated that edges involving the bridge symptoms were stronger than those involving other symptoms, although the strongest edges connected symptoms within the same disorder. The strongest cross-disorder edge between SMA and anxiety was BSMAS6–GAD5 at T1 and BSMAS5–GAD5 at T2 (**Supplementary Figure 5**). Edge weights and centrality stability for the networks at both time points met recommended thresholds (CS >.439; **Supplementary Table 1**, **Supplementary Figure 6**).

### 3.4 The BSMAS–PHQ comorbidity network

**Figure 3:**
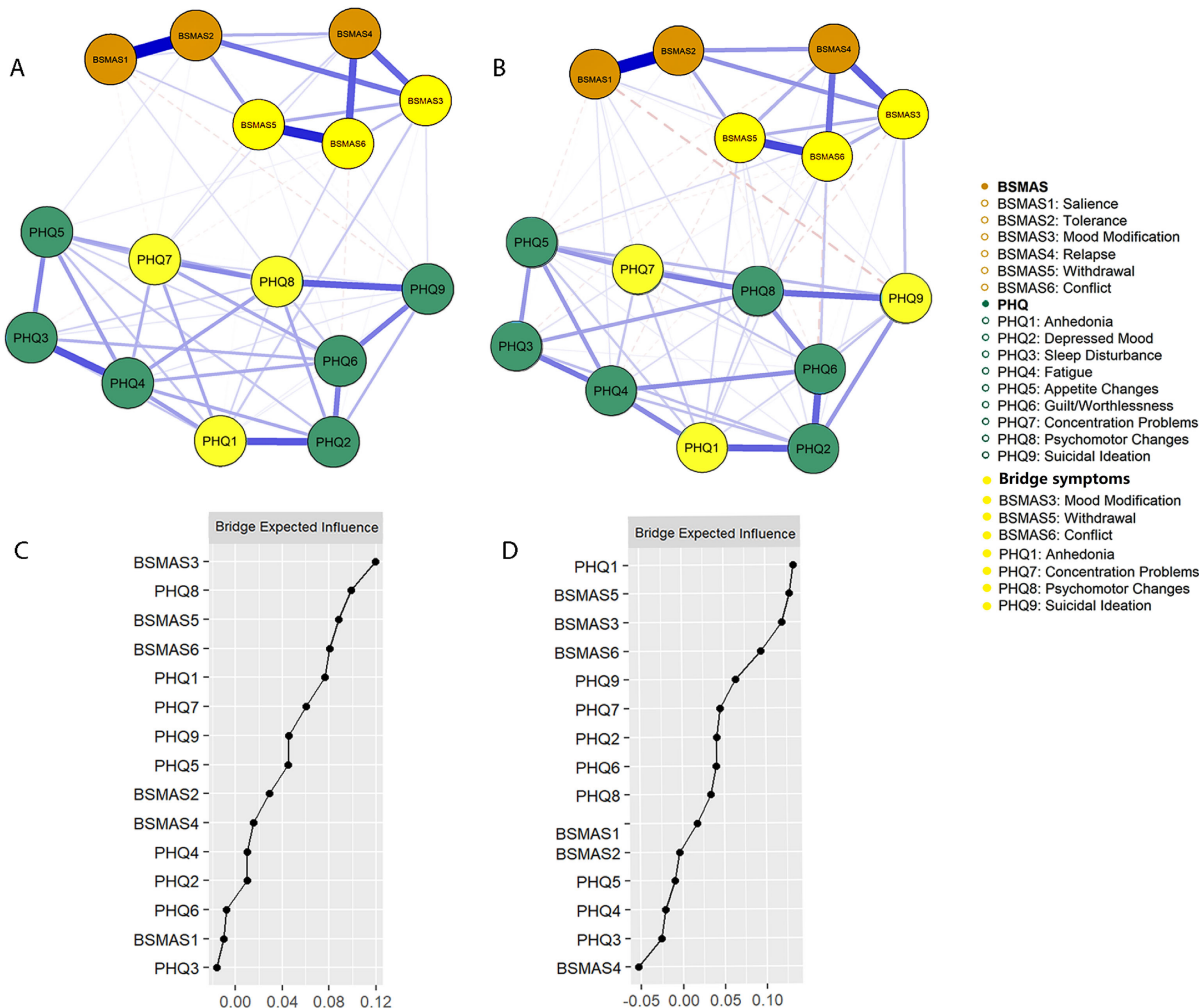
The comorbidity network structure and BEI plots for the BSMAS–PHQ network. The symptom networks for the BSMAS and the PHQ–9 at T1 **(A, C)** and T2 **(B, D)**. Orange nodes represent the BSMAS symptoms, green nodes represent the PHQ–9 symptoms, and yellow nodes indicate bridge symptoms. Edge thickness reflects partial correlations between symptoms; solid lines indicate positive associations, and dashed lines indicate negative associations. Node labels correspond to specific scale items as defined in the main text.

On the SMA side, the BSMAS3 (mood modification), BSMAS5 (withdrawal), and BSMAS6 (conflict) were consistently identified as bridging symptoms across both time points (**Figure 3**). For depressive symptoms, the PHQ8 (psychomotor changes), PHQ1 (anhedonia), and PHQ7 (concentration problems) at T1, and the PHQ1 (anhedonia), PHQ9 (suicidal ideation), and PHQ7 (concentration problems) at T2 were identified as bridging symptoms. Difference tests showed that the bridge symptoms, including BSMAS3, BSMAS5, BSMAS6, PHQ1, PHQ7, PHQ9, and PHQ8, had higher BEI than other symptoms at both T1 and T2; however, only the top five symptoms had significantly higher BEI than the lowest symptoms, and most symptoms did not differ significantly from each other in BEI (**Supplementary Figure 7**). Edge-weight difference tests produced similar findings to those in the BSMAS–GAD network: edges involving bridge symptoms were stronger than others, and the strongest edges occurred within the same disorder. The strongest cross-disorder edge in this network was BSMAS3–PHQ1 at T1 and BSMAS3–PHQ9 at T2 (**Supplementary Figure 8**). The edge weights and centrality stability of the networks met the recommended criteria, with CS >.439 (**Supplementary Table 1**, **Supplementary Figure 9**).

### 3.5 The integrated BSMAS–GAD–PHQ network

**Figure 4:**
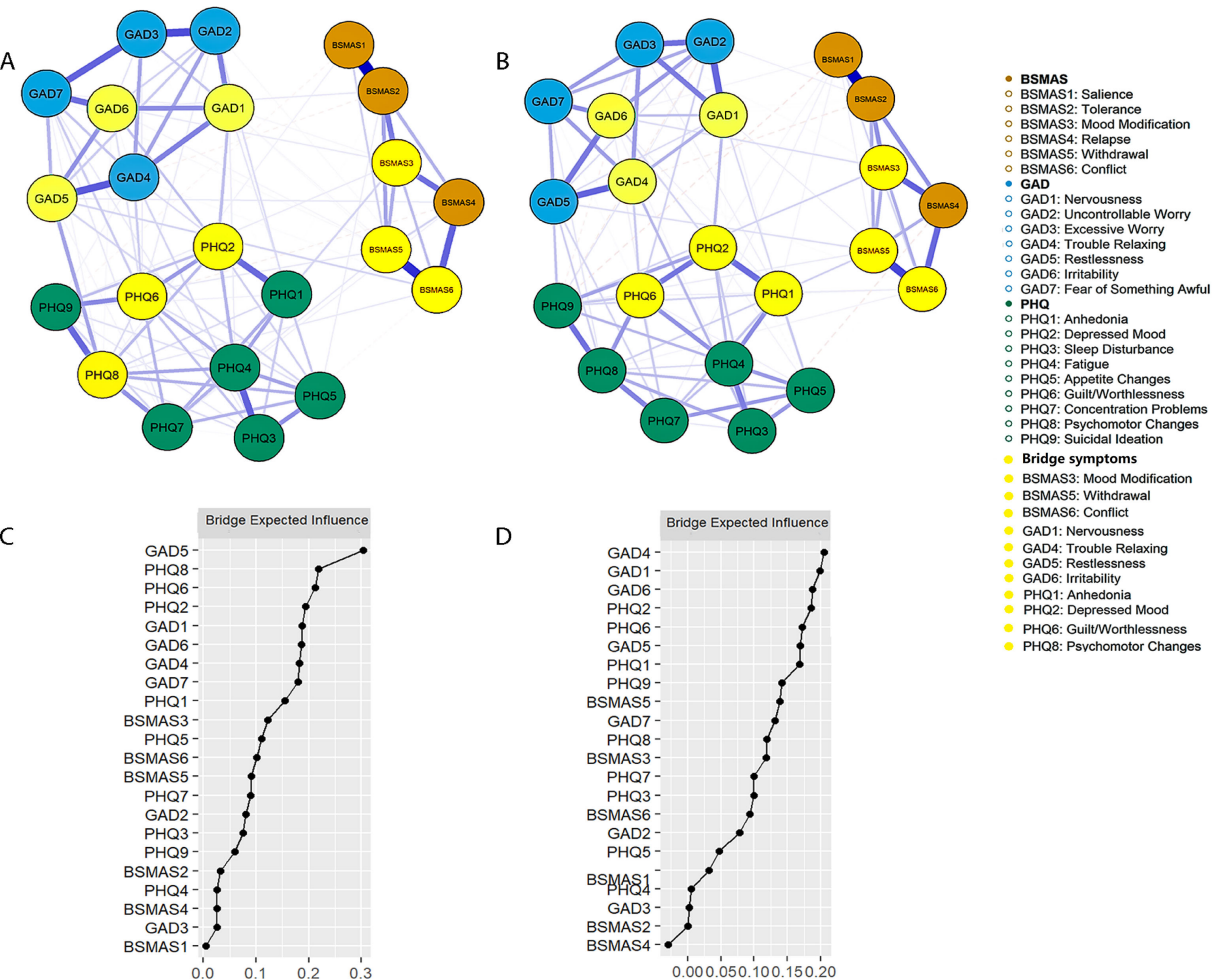
The comorbidity network structure and the BEI plots for the BSMAS–GAD–PHQ network. The symptom networks for the BSMAS, the GAD–7, and the PHQ–9 at T1 **(A, C)** and T2 **(B, D)**. Orange nodes represent the BSMAS symptoms, blue nodes represent the GAD–7 symptoms, green nodes represent the PHQ–9 symptoms, and yellow nodes indicate bridge symptoms. Edge thickness reflects partial correlations between symptoms; solid lines indicate positive associations, and dashed lines indicate negative associations. Node labels correspond to specific items as defined in the main text.

On the SMA side, consistent with the comorbidity networks, the BSMAS3 (mood modification), BSMAS5 (withdrawal), and BSMAS6 (conflict) were identified as bridge symptoms across both time points (**Figure 4**). Among anxiety symptoms, GAD5 (restlessness), GAD1 (nervousness), and GAD6 (irritability) were identified as bridging symptoms at T1, while GAD4 (trouble relaxing), GAD1 (nervousness), and GAD6 (irritability) were noted at T2. For depressive symptoms, PHQ8 (psychomotor changes), PHQ6 (guilt/worthlessness), and PHQ2 (depressed mood) were identified as bridging symptoms at T1, whereas PHQ2 (depressed mood), PHQ6 (guilt/worthlessness), and PHQ1 (anhedonia) were identified at T2. Difference tests indicated that bridge symptoms exhibited significantly higher BEI than other symptoms, particularly compared with symptoms within their own communities (see **Supplementary Figure 10**). Edge-weight difference tests revealed a similar connection pattern to the comorbidity networks: edges involving bridge symptoms were stronger than others, and the strongest edges occurred within the same disorder. The strongest SMA-related cross-disorder edge was BSMAS6–GAD5, followed by BSMAS3–PHQ1 at T1; at T2, the strongest SMA-related cross-disorder edge was BSMAS3–PHQ9, followed by BSMAS5–PHQ1 and BSMAS3–GAD6 (**Supplementary Figure 11**). Both BEI and edge weights showed excellent stability, with CS >.594 (**Supplementary Table 1**, **Supplementary Figure 12**).

### 3.6 Network temporal stability

**Table 3** presents the correlations between the corresponding network metrics at T1 and T2. Across all networks, edge weights (*r* = .892 –.973, all *p* <.001), the EI (*r* = .806 –.961, all *p* ≤.002), and the BEI values (*r* = .699 –.804, all *p* ≤.008) all demonstrated high stability across the two assessments, suggesting substantial temporal stability of node-level metrics. Moreover, the NCT analysis revealed no significant changes in overall network structures from T1 to T2 for all networks (all *p* >.05; **Table 4**), indicating robust topological consistency of the network structures. NCT also detected significant changes in global strength across time for all four networks (all *p* ≤.005). Notably, however, these changes were primarily driven by three edges, BSMAS2 (tolerance)–BSMAS4 (relapse), BSMAS1 (salience)–BSMAS4 (relapse), and BSMAS3 (mood modification)–BSMAS4 (relapse), whereas the other edges remained stable (**Supplementary Table 2**). Together, these results support the temporal stability of network structure and node-level metrics across all four networks.

**Table 3:** Correlations between network metrics at T1 and T2.

Network	Metric	r	95% CI	p
BSMAS	Edge weights	.973	.944 –.987	<.001
	EI	.961	.681 –.996	.002
BSMAS–GAD	Edge weights	.908	.876 –.932	<.001
	EI	.806	.460 –.940	<.001
	BEI	.699	.240 –.902	.008
BSMAS–PHQ	Edge weights	.900	.871 –.923	<.001
	EI	.939	.822 –.980	<.001
	BEI	.715	.319 –.898	.003
BSMAS–GAD–PHQ	Edge weights	.892	.872 –.909	<.001
	EI	.903	.777 –.959	<.001
	BEI	.804	.578 –.915	<.001

*r*, Pearson’s correlation coefficient across T1 and T2. 95% confidence intervals (CIs) were estimated via Fisher’s *z*-transformation. All *p-values* are two-tailed.

**Table 4:** Results of the network comparison test between T1 and T2.

Network	Network structure invariance statistic	*p*	Global strength invariance statistic	*p*
BSMAS	0.072	.925	0.093	.001
BSMAS–PHQ	0.133	.879	0.109	.004
BSMAS–GAD	0.172	.469	0.089	.003
BSMAS–GAD–PHQ	0.166	.704	0.112	.005

The network structure invariance statistic indicates the difference in overall network structure between T1 and T2, and the global strength invariance statistic indicates the difference in overall connectivity strength between T1 and T2. All *p-values* are two-tailed.

## 4 Discussion

This study was carried out to investigate the temporal stability of core SMA symptoms and the bridging symptoms with anxiety and depression. The key findings included: 1) Although SMA, anxiety, and depression levels of respondents rose significantly over the year, all four networks showed strong temporal stability, with the edge weights (*r* = .892 –.973, *p* <.001), the EI (*r* = .806 –.961, *p* ≤.002), and the BEI (*r* = .699 –.804, *p* ≤.008) highly correlated between T1 and T2; network comparison tests showed no significant changes in overall structures of all four networks, with most edges showing stable strength. 2) Within the BSMAS network, BSMAS2 (tolerance) and BSMAS6 (conflict) exhibited the highest EI at both time points. 3) In the comorbidity networks, BSMAS3 (mood modification), BSMAS5 (withdrawal), and BSMAS6 (conflict) consistently served as bridge symptoms on the SMA side at both T1 and T2. 4) Across both time points, PHQ1 (anhedonia) and PHQ7 (concentration problems) exhibited the highest BEI on the depression side, whereas GAD1 (nervousness) and GAD5 (restlessness) did so on the anxiety side. 5) These bridge symptoms were also confirmed in the integrated network.

### 4.1 The overall associations between SMA, anxiety, and depression

This study found that levels of SMA, anxiety, and depression were significantly increased at the follow-up survey, suggesting a worsening risk for these conditions, consistent with earlier findings (53, 54). Despite the increased levels of symptoms, the current study demonstrated moderate-to-very high mutual correlations between scale scores on SMA, depression, and anxiety symptoms at both T1 and T2 (*r* = .389 –.748, all *p* <.001; **Table 2**), indicating close associations among these conditions. These results were consistent with previous meta-analyses suggesting a close association between these disorders (55, 56).

### 4.2 SMA core symptoms

Within the BSMAS network, items 2 (Tolerance) and 6 (Conflict) exhibited the highest EI scores among all SMA symptoms at both time points. These results were consistent with previous studies by Tullett-Prado et al. (30) and Li et al. (27) in which Tolerance was identified as a core symptom of SMA. These findings are also consistent with the component model of addiction, in which conflict is regarded as a central component of behavioral addiction (57, 58). Moreover, consistent with our results, a recent longitudinal network analysis indicated that both BSMAS2 (Tolerance) and BSMAS6 (Conflict) consistently occupied central positions across different time points (30). Tolerance is a diagnostic symptom of various substance and behavioral addictions, referring to the increasing need for escalated engagement with the addictive stimulus to achieve the same psychological effect (59, 60). Such escalation may gradually weaken self-regulatory functioning, making it more difficult to disengage from social media. Crucially, unlike in other addictions, tolerance in SMA may exert a broad influence on users’ lives, because social media is deeply embedded in everyday communication, social bonding, and emotion regulation, making it a fundamental symptom of SMA (61–63). Conflict is particularly significant as it captures the degree to which excessive social media use disrupts offline functioning. Our results suggest that the interaction between tolerance and conflict may be central to the maintenance and development of SMA in adolescents. Excessive social media use (tolerance) can reduce family interaction, provoke disputes about screen time, and elicit feelings of criticism, thereby escalating family conflict. That conflict can, in turn, worsen depressive mood and anxiety-related distress. Thus, conflict is not only a consequence of SMA but may also operate as a key mechanism linking SMA with depression and anxiety. In support of this perspective, BSMAS6 (conflict) was consistently identified as a bridge symptom in the BSMAS–GAD and BSMAS–PHQ comorbidity networks, and the BSMAS6–GAD5 edge emerged as one of the strongest cross-disorder connections.

### 4.3 Bridge mechanisms linking SMA with depression and anxiety

In the comorbidity networks, BSMAS3 (mood modification), BSMAS5 (withdrawal), and BSMAS6 (conflict) consistently emerged as key bridge symptoms linking SMA with depressive and anxiety symptoms, with the BSMAS6–GAD5 (restlessness) and BSMAS3–PHQ1 (anhedonia) as the strongest cross-disorder edges. These findings closely align with previous cross-sectional network studies, such as Lin et al. (64) and Li et al. (27), which identified these bridging symptoms linking SMA with depression, anxiety, and related negative emotional states. A recent network analysis by Peng and Liao (65) also highlighted that Conflict functioned as a bridge symptom between SMA and psychological distress (e.g., depression, anxiety, and stress). Withdrawal and Conflict served as bridge nodes connecting different types of addictive behaviors. Notably, when SMA was restricted, adolescents exhibited heightened emotional withdrawal responses, which, if prolonged, could further impair real-life functioning. Aligned with our results, Tullett-Prado et al. (30) identified mood modification and conflict as core bridging symptoms connecting SMA with anxiety and stress, while also emphasizing the mediating role of withdrawal symptoms. They proposed a potential pathway whereby adolescents begin using social media to cope with negative emotions, which then leads to functional impairments such as interpersonal conflict, creating new sources of emotional distress. This pattern of cyclical reinforcement between psychological distress and SMA mirrors the maintenance mechanisms observed in other behavioral addictions (66, 67). These results were also consistent with the I-PACE model, which highlights the fundamental role of negative emotions and maladaptive emotion regulation strategies in the maintenance and development of behavioral addictions (37).

Moreover, this study showed that the bridging pattern remained stable across both comorbidity networks between SMA and depression and SMA with anxiety, even when all three symptom clusters were considered simultaneously in the BSMAS–GAD–PHQ network. Together, our results expand these findings by suggesting a consistent and temporally stable bridging role of mood modification, withdrawal, and conflict toward emotional symptoms in adolescents. Taken together, these findings support a vicious cycle in which adolescents use social media to cope with negative emotions, increased use strengthens mood modification, withdrawal, and conflict, and these consequences in turn intensify depression and anxiety, thereby promoting further SMA. Note that, however, we excluded participants with a history of major mental disorders, including depressive and anxiety disorders. Therefore, the mechanisms described above may differ in more severe, long-standing cases: in individuals with chronic or severe depression or anxiety, symptom dynamics and bridge pathways could be altered, amplified, or operate through additional mechanisms not captured in our sample.

### 4.4 Bridge symptoms on the anxiety and depression side

In the comorbidity network of BSMAS–GAD, we found that GAD1 (nervousness) and GAD5 (restlessness) consistently showed high BEI on the anxiety side. These findings are in line with a recent study of internet-related addictive behaviors, which highlighted GAD1 and GAD5 as important nodes linking anxiety and addiction symptoms (68). Similarly, Peng and Liao (65) reported that restlessness often occupied a bridging position in symptom networks and was positively related to addiction features such as conflict and withdrawal. Such symptoms may heighten physiological arousal and sensitivity to external stimuli, thereby increasing the likelihood of anxiety–addiction comorbidity. From a mechanistic perspective, nervousness and restlessness may exert their bridging effect through the process of fear of missing out (FOMO). FOMO has been conceptualized as an anxiety-related social cognitive factor that drives individuals to check and browse social media repeatedly in order to avoid missing important updates or interactions (69). In particular, FOMO has been identified as an important mediator that links psychological distress to maladaptive usage patterns (70). Taken together, these findings suggest that nervousness and restlessness may increase sensitivity to social cues and FOMO, thereby intensifying SMA-related motivations and behaviors.

In the comorbidity network of BSMAS–PHQ, PHQ1 (Anhedonia) and PHQ7 (Concentration problems) showed the highest BEI on the depression side in both time points. Our findings are in line with previous findings that “concentration problems” in depression acted as a bridge symptom linking problematic smartphone use (71). Likewise, previous evidence indicates that Anhedonia can predict compulsive internet use and increased risk of addiction (72). Recent network analysis by Cai et al. (73) identified Anhedonia as a key bridge symptom connecting depression and Internet addiction. The impact of anhedonia and concentration problems on SMA may be understood through two complementary theoretical perspectives. First, according to the Reward Deficiency Syndrome model (74), individuals with depression experience blunted neural responses to natural rewards. To compensate for this deficit, they may seek stronger external stimuli—such as social media interactions—which can paradoxically increase the risk of addictive behaviors. Recent research supports applying this framework to digital environments, suggesting that impaired reward processing drives increased engagement with online interactions as substitute reinforcers (75). Second, concentration problems reflect impaired cognitive control in depression. Hoorelbeke et al. (76) highlighted the central role of impaired attentional control in depression, showing that deficits in attentional regulation are closely linked to difficulties in emotion regulation. These findings suggest that compromised attentional control increases vulnerability to external cues—including social media stimuli—thereby setting up a feedback loop of attentional problems, excessive engagement, and functional impairment that could ultimately contribute to the development of SMA.

### 4.5 Temporal stability of the networks

Crucially, our results identified the same core symptoms and bridge symptoms in both the BSMAS network and the comorbidity networks in two surveys administered one year apart, indicating a significant temporal stability of influential symptoms in SMA and the interaction of symptoms between disorders.

For the BSMAS network, both centrality (EI, *r* = .961, *p* ≤.002) and edge weights (*r* = .973, *p* <.001) showed high correlations between T1 and T2, suggesting that the relative importance of nodes was broadly consistent over time. Our NCT analysis also confirmed the temporal stability of the overall topological consistency of the network, by indicating no significant change between the two time points. Our results were consistent with previous longitudinal network analysis, where identical core symptoms have been recognized in multiple waves of surveys (30, 77). Together, these findings suggest that interrelationships between symptoms within a disorder could be highly stable across time.

We identified the same bridge symptoms across time, particularly on the BSMAS side. In the comorbidity networks, BSMAS3 (mood modification), BSMAS5 (withdrawal), and BSMAS6 (conflict) consistently emerged as key bridge symptoms for SMA at both T1 and T2. On the depression and anxiety sides, PHQ1 (anhedonia), PHQ7 (concentration problems), GAD1 (nervousness), and GAD5 (restlessness) consistently showed the highest bridge BEI. The strongest cross-disorder edges were BSMAS6–GAD5 (restlessness) and BSMAS3–PHQ1 (anhedonia). Importantly, these results were corroborated in the integrated network that included all SMA, anxiety, and depression symptoms, and by NCT analyses showing that the overall network structure remained stable between T1 and T2. The findings of significant similarity between the T1 and T2 networks were in line with previous longitudinal network studies (30, 77), suggesting a robust temporal stability in network structure.

Notably, mean levels of SMA, depression, and anxiety increased in our sample. Despite this rise in severity at the group level, the stability of core and bridge symptoms supports Jones and Robinaugh’s (78) contention that symptom network structure tends to persist over time. In other words, symptom interrelations within individuals showed continuity, suggesting structural vulnerability or persistence rather than transient fluctuations. This temporal stability applied to both global network organization and influential local nodes, implying that the relationships among SMA, depression, and anxiety symptoms reflect enduring vulnerability structures. In particular, bridge symptoms that link SMA and affective symptoms may serve as stable conduits for transmitting distress across domains—consistent with longitudinal network studies highlighting the persistence of bridge pathways (79, 80).

## 5 Limitations

One notable limitation of this study is the gender imbalance in the sample, which is overtly skewed toward girls. We considered this acceptable given evidence that females are more likely to use social media as a means of emotion regulation (including anxiety and depression) and, as a result, may be more susceptible to SMA (39, 81, 82). Nonetheless, studies using gender-balanced samples are needed to provide a more complete picture of comorbidity patterns among social media addiction, depression, and anxiety. In addition, the sample was drawn from a single school in western China. The relative homogeneity of the geographic and cultural background may limit the generalizability of our findings. Replications with more diverse and cross-cultural samples are needed to further validate our findings. Additionally, we excluded participants with a history of any mental disorders to minimize confounding from severe psychiatric conditions and from variability in treatment exposure. However, this sampling strategy also constrained our sample to a relatively healthy, school-based population and excluded adolescents at the more severe end of the spectrum. Consequently, the core and bridging symptoms identified here should be interpreted with caution and should not be directly generalized to adolescents with clinically significant psychiatric disorders. Finally, the study included only two measurement occasions, which limited our ability to detect fine-grained temporal dynamics among SMA, depression, and anxiety symptoms. Future research would benefit from higher-frequency longitudinal designs to more precisely capture and clarify the complex temporal interactions among these symptoms.

## 6 Conclusions

The findings of this study align with the theoretical perspective proposed by Epskamp and colleagues (83), which highlights the significance of temporal connections between symptoms for identifying effective intervention targets. This perspective provides a theoretical foundation for developing temporally sensitive psychological intervention strategies.

Across the two time points, “tolerance” (BSMAS2) and “conflict” (BSMAS6) consistently exhibited high centrality, indicating their stable and prominent roles within the cross-temporal structure of SMA. In addition, “conflict” (BSMAS6), “mood modification” (BSMAS3), and “withdrawal” (BSMAS5) persistently served as bridging symptoms between SMA and anxiety–depression symptomatology, representing potential pathways for comorbidity transmission. The temporal stability of these bridge symptoms suggests the existence of a pulling effect (23), whereby the activation of these nodes may trigger cascades of emotional symptoms over time.

Clinically, our results suggest that targeting “conflict” and “tolerance” may help disrupt the internal maintenance mechanisms of SMA, while early detection and intervention on bridge symptoms such as “mood modification”, “conflict”, and “withdrawal” may interrupt the transmission channel between SMA and emotional disorders. This approach could enhance the efficiency of comorbidity prevention and contribute to the development of more targeted and effective intervention frameworks (84).

In sum, through a two-wave longitudinal network analysis, this study identified both core and bridging symptoms in the comorbidity network of SMA, depression, and anxiety. The results reveal that “tolerance” and “conflict” play consistently central roles within the symptom network of SMA, while “mood modification,” “conflict,” and “withdrawal” function as key bridges linking SMA with emotional distress. Compared to cross-sectional designs, this longitudinal approach sheds light on the temporal stability and evolution of symptom architecture, offering a more dynamic perspective on the comorbidity of SMA, depression, and anxiety in adolescents.

## Data Availability

The raw data supporting the conclusions of this article will be made available by the authors, without undue reservation.
