# Supplementary material for: The temporal stability of core symptoms of social media addiction and their comorbidity with anxiety and depression in adolescents: a longitudinal network analysis
Source: Front Psychiatry. 2026 Apr 22;17:1785472. doi: 10.3389/fpsyt.2026.1785472 (PMC13144121; doi:10.3389/fpsyt.2026.1785472)
Supplement: Supplementary file 1 [file Supplementaryfile1.docx]

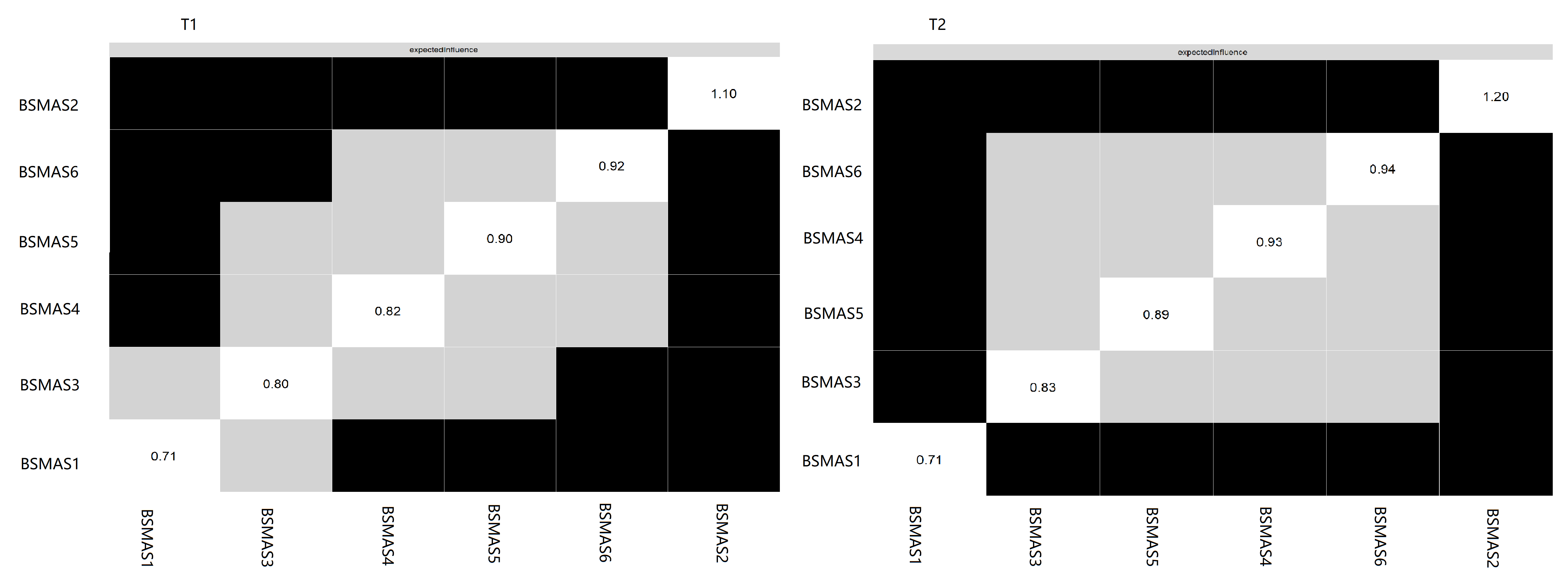
**Supplementary materials**


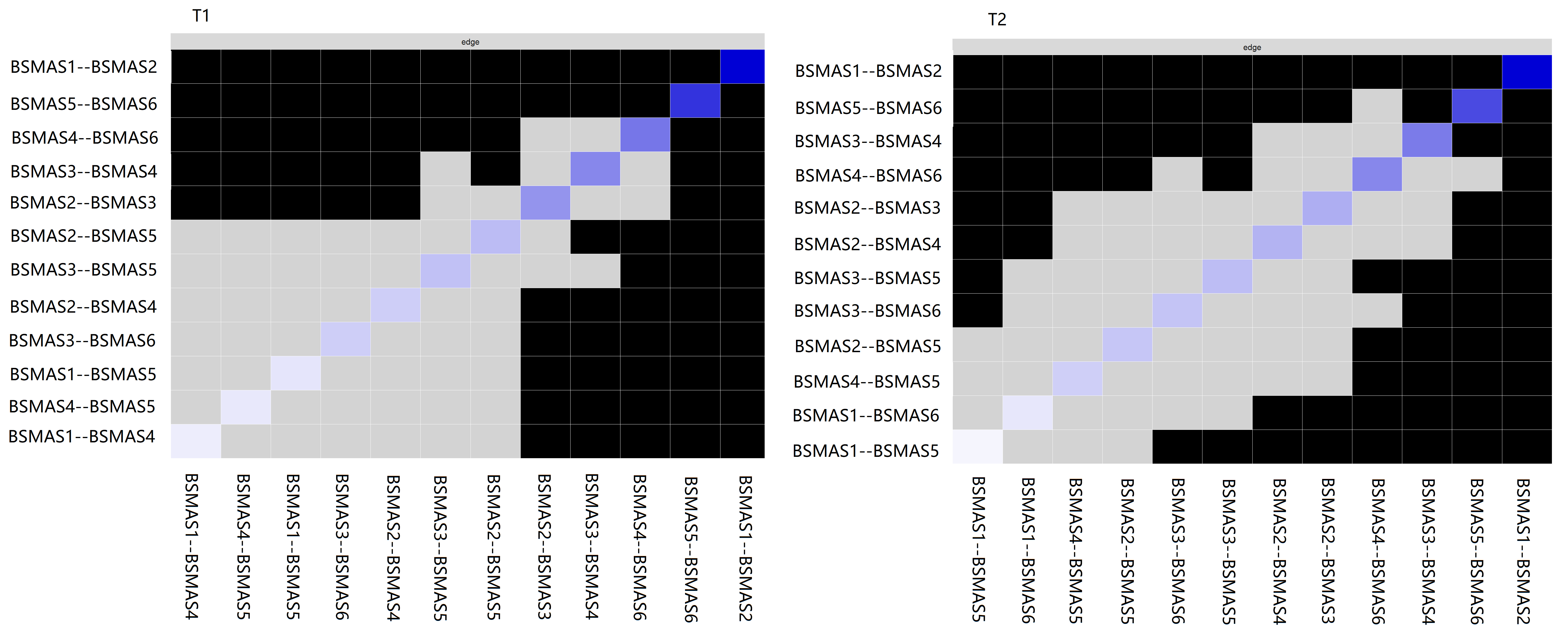
**Figure S1**. *Bootstrapped difference test for node EI in the BSMAS networks*

**Figure S2**. *Bootstrapped difference test for edge weights in the BSMAS networks*


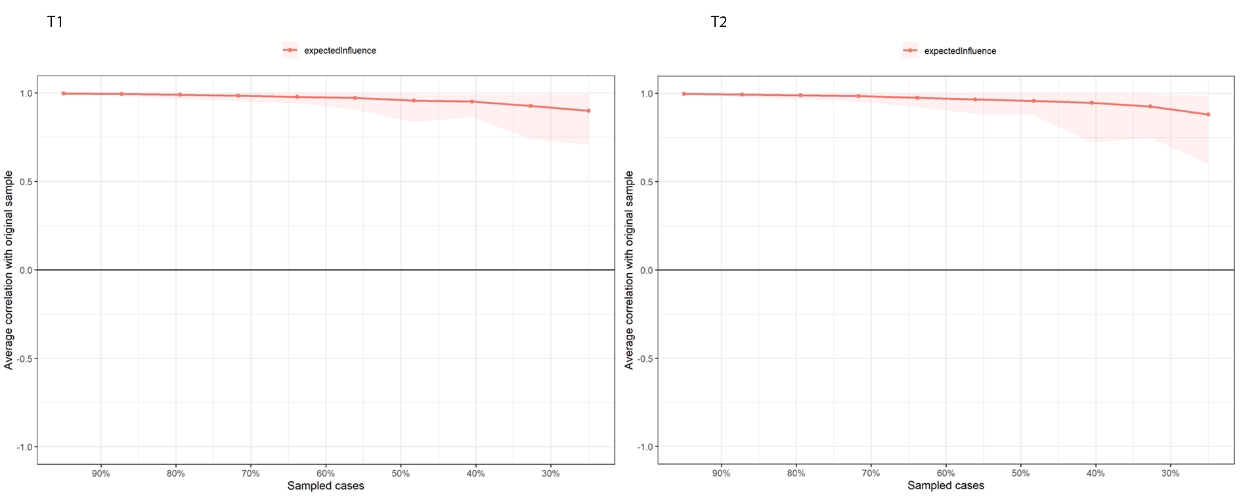


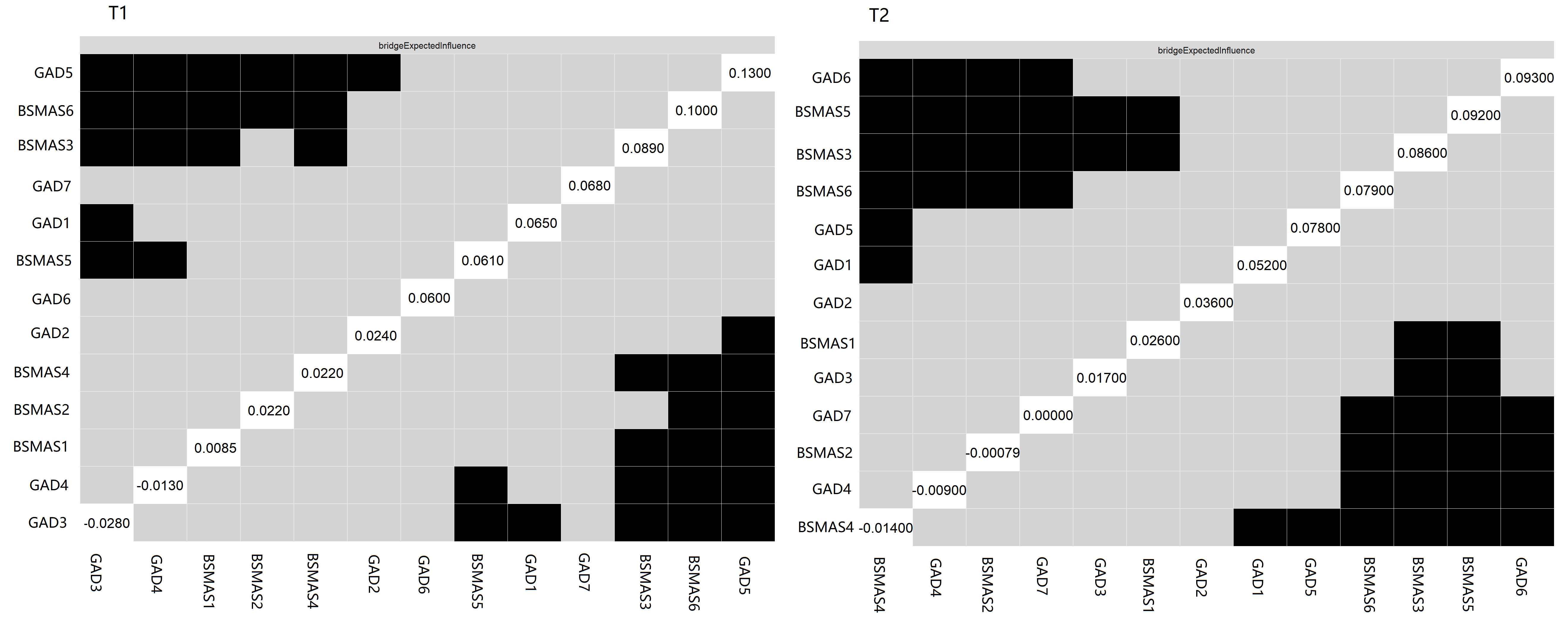
**Figure S3.** *CS-coefficient plots for the BSMAS networks*

**Figure S4**. *Bootstrapped difference test for BEI in the BSMAS–GAD network*


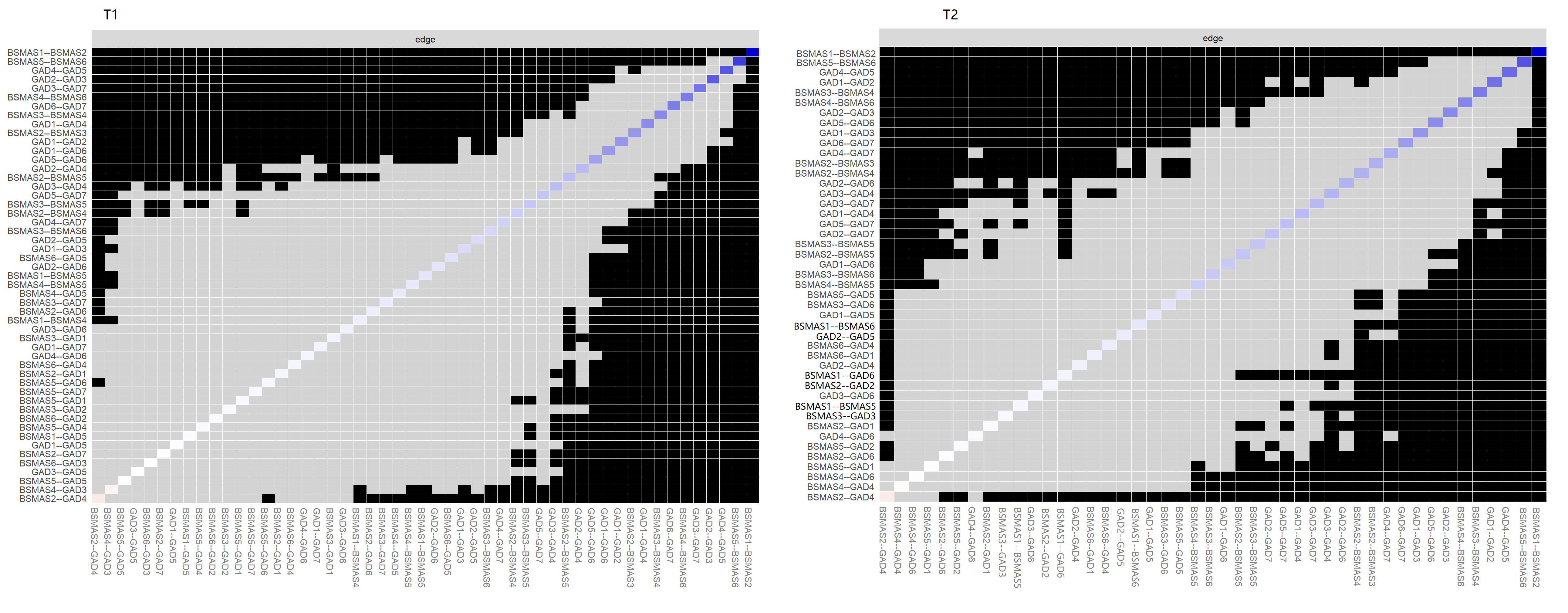


**Figure S5**. *Bootstrapped difference test for edge weights in the BSMAS–GAD networks*


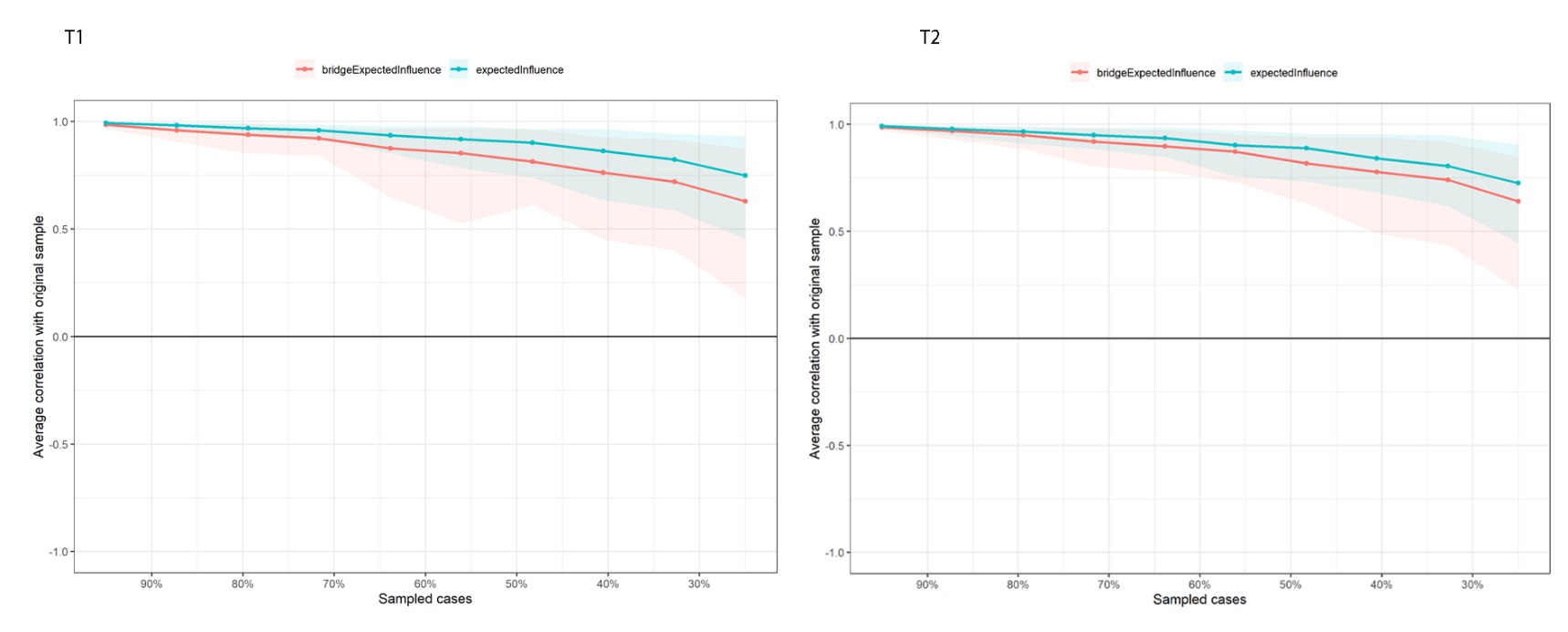


**Figure S6.** *CS-coefficient plots for the BSMAS*–*GAD networks*


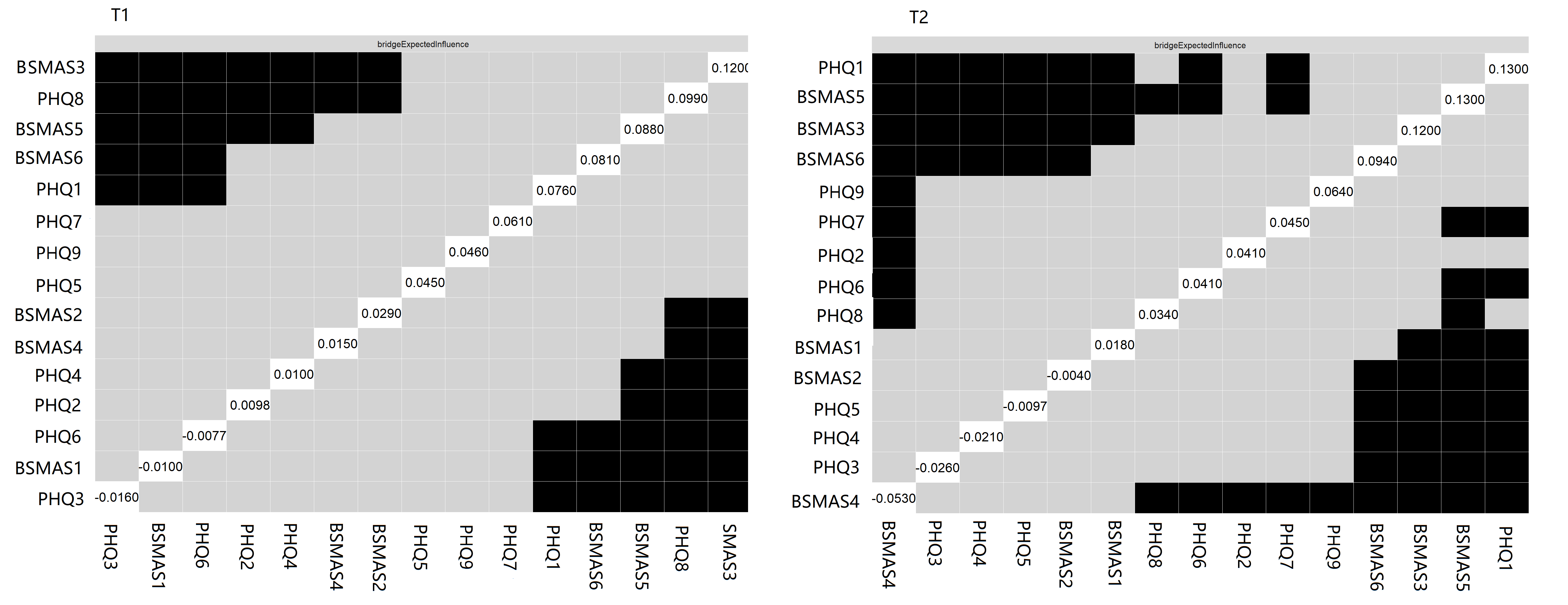


**Figure S7**. *Bootstrapped difference test for BEI in the BSMAS–PHQ networks*


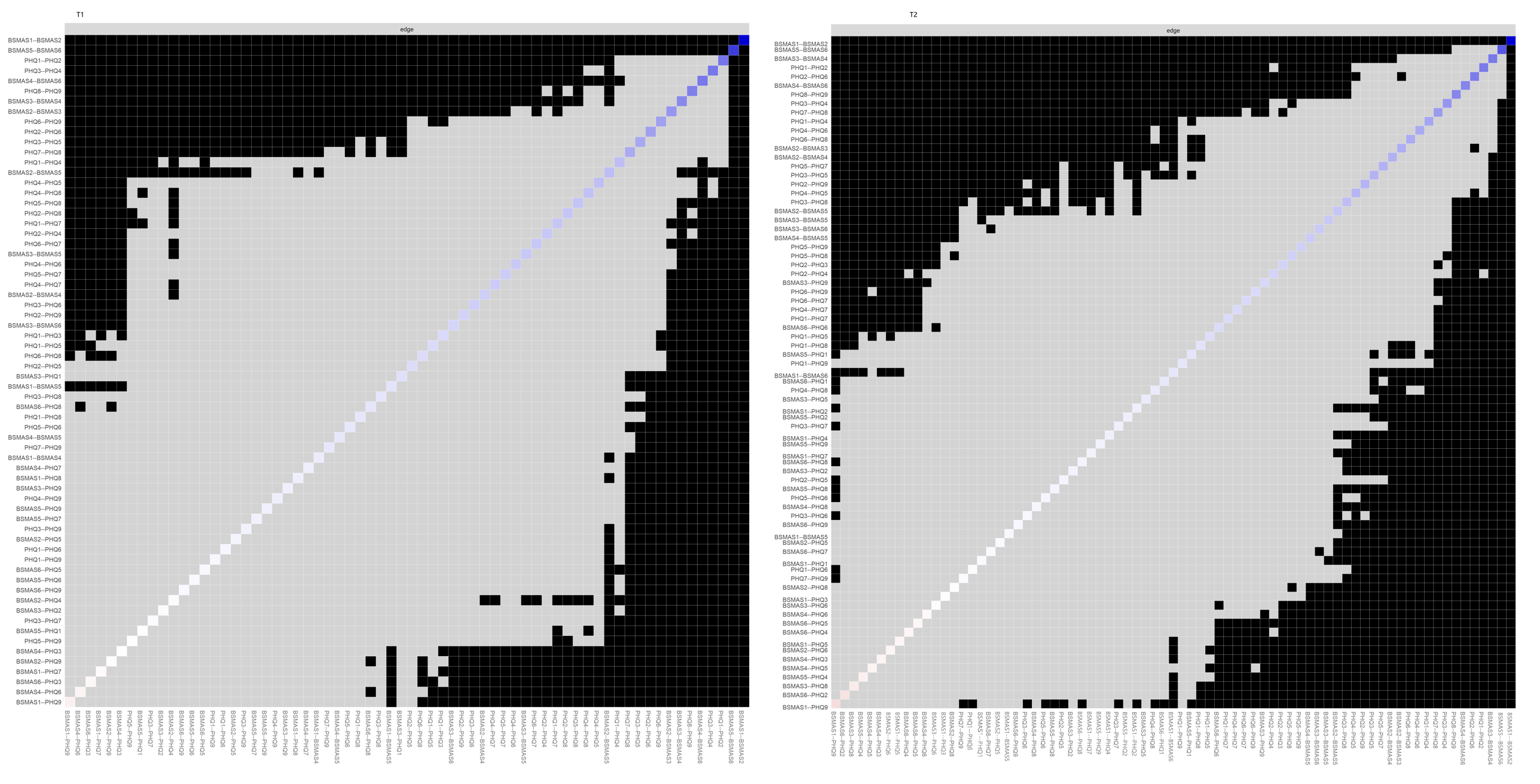


**Figure S8**. *Bootstrapped difference test for edge weights in the BSMAS–PHQ networks*


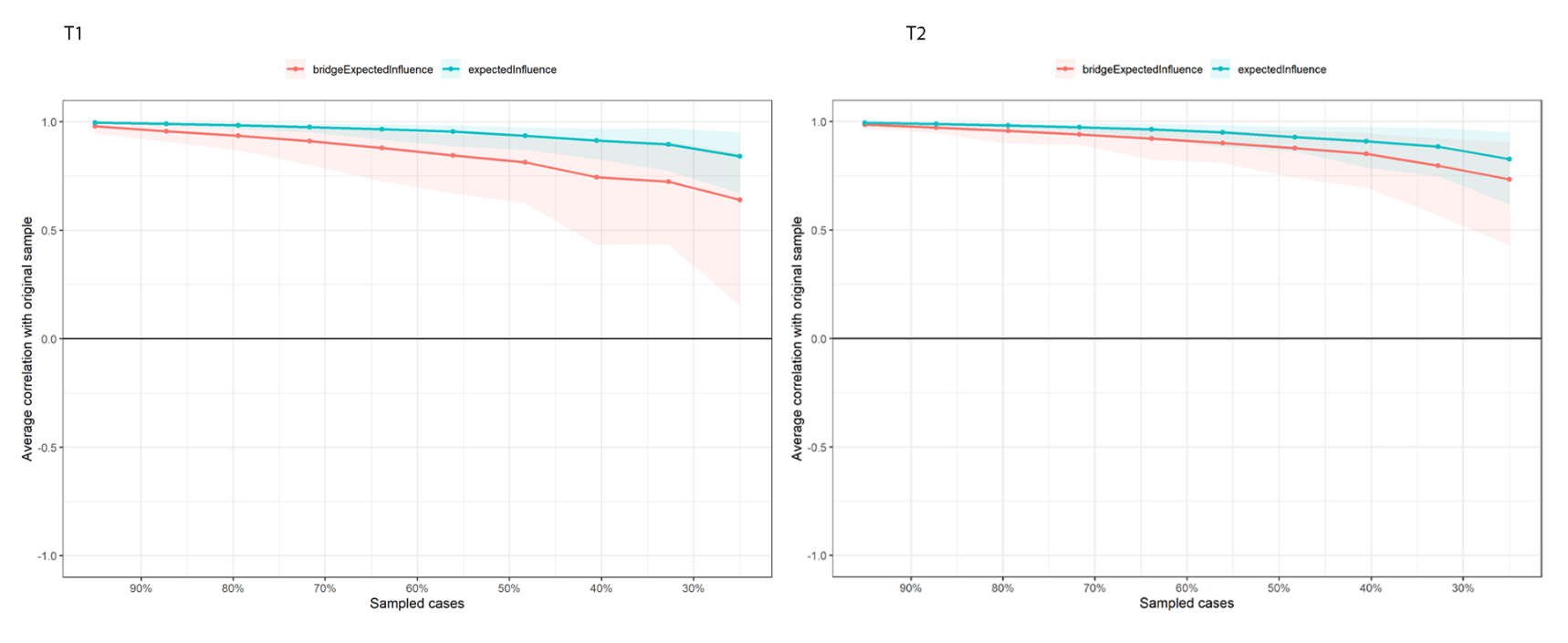


**Figure S9.** *CS-coefficient plots for the BSMAS*–*PHQ* *networks*


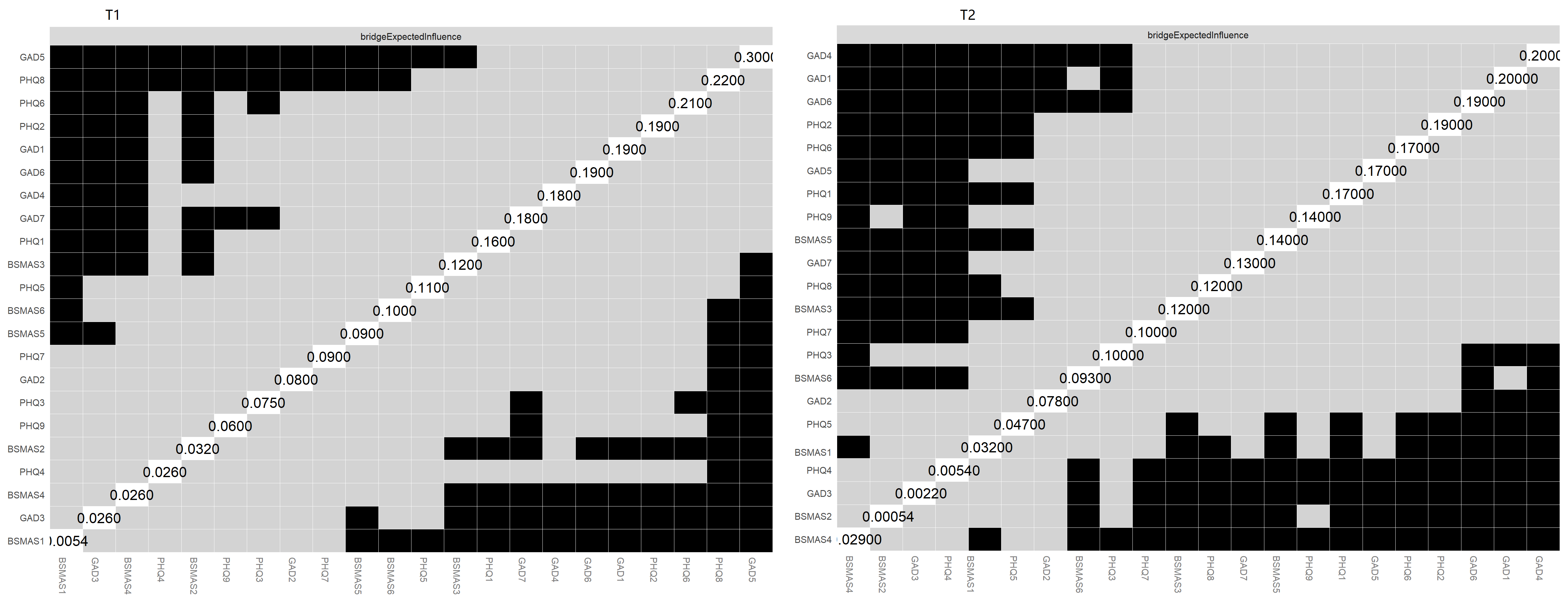
**Figure S10.** *Bootstrapped difference test for BEI in the BSMAS–GAD–PHQ networks*


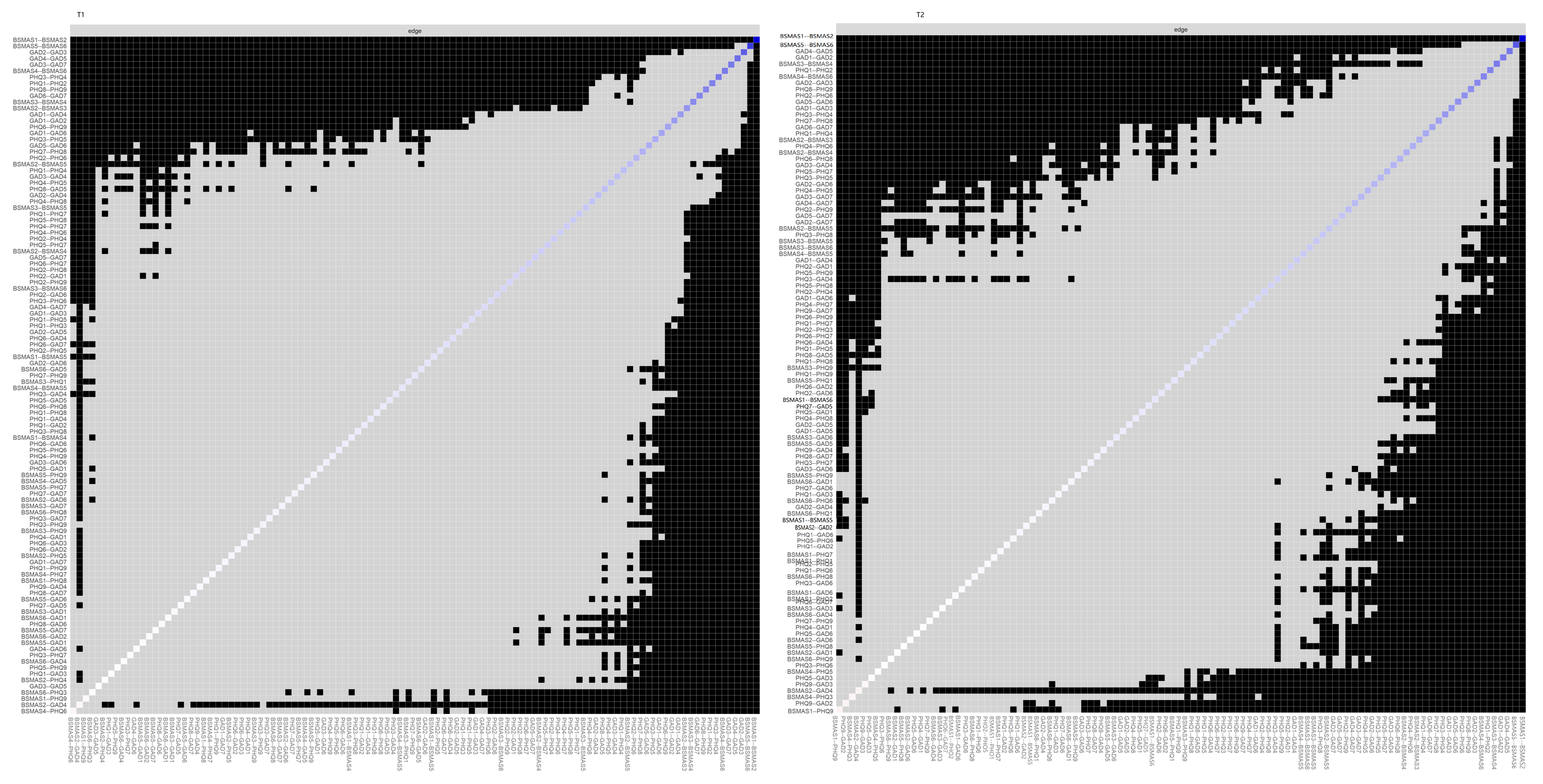


**Figure S11**. *Bootstrapped difference test for edge weights in the BSMAS–GAD–PHQ networks*


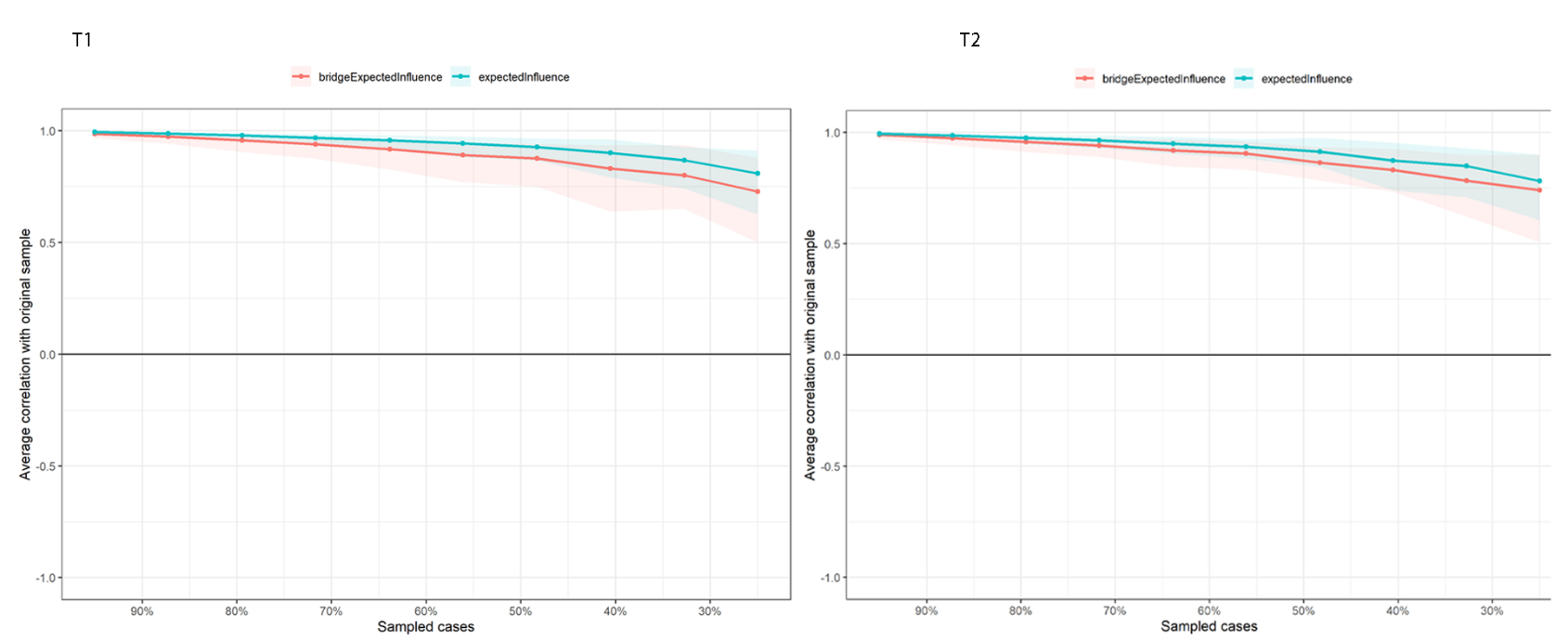


**Figure S12.** *CS-coefficient plots for the BSMAS*–*GAD*–*PHQ networks*

**Table S1.** *Summary of CS-coefficient values for EI and BEI across all networks*

| Network | Metric | CS-Coefficient |
| --- | --- | --- |
| T1 BSMAS | EI | .750 |
| T2 BSMAS | EI | .750 |
| T1 BSMAS–GAD | EI | .673 |
|  | BEI | .439 |
| T2 BSMAS–GAD | EI | .594 |
|  | BEI | .439 |
| T1 BSMAS–PHQ | EI | .750 |
|  | BEI | .439 |
| T2 BSMAS–PHQ | EI | .750 |
|  | BEI | .594 |
| T1 BSMAS–GAD–PHQ | EI | .673 |
|  | BEI | .594 |
| T2 BSMAS–GAD–PHQ | EI | .673 |
|  | BEI | .594 |

Note: EI, Expected Influence; BEI, Bridge Expected Influence.

**Table S2**. *Significant edge differences between T1 and T2 across all networks.*

| Network | Edge | Weight at T1 | Weight at T2 | Difference  (T2–T1) | *p* |
| --- | --- | --- | --- | --- | --- |
| BSMAS | Tolerance-Relapse | .116 | .188 | 0.072 | < .001 |
|  | Salience-Relapse | .043 | .000 | -0.043 | .024 |
|  |  |  |  |  |  |
| BSMAS–PHQ | Tolerance-Relapse | .117 | .192 | 0.076 | < .001 |
|  | Salience-Relapse | .168 | .122 | -0.046 | .023 |
|  | Mood Modification-Relapse | .071 | .119 | 0.048 | .047 |
|  |  |  |  |  |  |
| BSMAS–GAD | Tolerance-Relapse | .118 | .191 | 0.073 | < .001 |
|  | Salience-Relapse | .164 | .121 | -0.043 | .021 |
|  |  |  |  |  |  |
| BSMAS–GAD–PHQ | Tolerance-Relapse | .115 | .190 | 0.075 | < .001 |
|  | Salience-Relapse | .166 | .121 | -0.045 | .029 |
|  | Mood Modification-Relapse | .067 | .120 | 0.053 | .046 |

Note: Remaining edges (not listed) showed no significant T1→T2 changes. Positive values indicate increased edge strength from T1 to T2, negative values indicate decreased. All *p-values* are two-tailed.
